# Supplementary material for: Modeling glioblastoma heterogeneity as a dynamic network of cell states
Source: Mol Syst Biol. 2021 Sep 16;17(9):e10105. doi: 10.15252/msb.202010105 (PMC8444284; doi:10.15252/msb.202010105)
Supplement: Supplementary file 6 — Source Data for Figure 5 [file MSB-17-e10105-s004.zip › Figure5A_sourcedata/GSEA_3017/nosigdb_stateB.GseaPreranked.1622795021725/RICHARDS_NOWAKOWSKI_SCIENCE_2017_IPC_NEN3_UPREG.html]

Details for gene set RICHARDS\_NOWAKOWSKI\_SCIENCE\_2017\_IPC\_NEN3\_UPREG[GSEA]

|  || Dataset | state43017 |
| Phenotype | NoPhenotypeAvailable |
| Upregulated in class | na\_neg |
| GeneSet | RICHARDS\_NOWAKOWSKI\_SCIENCE\_2017\_IPC\_NEN3\_UPREG |
| Enrichment Score (ES) | -0.6111901 |
| Normalized Enrichment Score (NES) | -3.4634354 |
| Nominal p-value | 0.0 |
| FDR q-value | 0.0 |
| FWER p-Value | 0.0 |
Table: GSEA Results Summary

  

Fig 1: Enrichment plot: RICHARDS\_NOWAKOWSKI\_SCIENCE\_2017\_IPC\_NEN3\_UPREG      
 Profile of the Running ES Score & Positions of GeneSet Members on the Rank Ordered List

  

| PROBE | GENE SYMBOL | GENE\_TITLE | RANK IN GENE LIST | RANK METRIC SCORE | RUNNING ES | CORE ENRICHMENT || 1 | SDC2 |  |  | 107 | 0.513 | -0.0893 | No |
| 2 | SYNE2 |  |  | 203 | 0.413 | -0.1733 | No |
| 3 | SLC1A3 |  |  | 237 | 0.388 | -0.1758 | No |
| 4 | HMGN2 |  |  | 243 | 0.386 | -0.1406 | No |
| 5 | EZR |  |  | 403 | 0.307 | -0.3229 | No |
| 6 | PPME1 |  |  | 423 | 0.299 | -0.3161 | No |
| 7 | PAX6 |  |  | 617 | 0.261 | -0.5496 | No |
| 8 | HEG1 |  |  | 639 | 0.257 | -0.5501 | No |
| 9 | RPL13A |  |  | 685 | -0.260 | -0.5829 | Yes |
| 10 | RPS25 |  |  | 687 | -0.263 | -0.5556 | Yes |
| 11 | MSMO1 |  |  | 691 | -0.267 | -0.5307 | Yes |
| 12 | GDI2 |  |  | 697 | -0.274 | -0.5076 | Yes |
| 13 | CA12 |  |  | 698 | -0.274 | -0.4778 | Yes |
| 14 | RPL23 |  |  | 704 | -0.283 | -0.4538 | Yes |
| 15 | CHD7 |  |  | 706 | -0.284 | -0.4242 | Yes |
| 16 | MYO10 |  |  | 710 | -0.305 | -0.3950 | Yes |
| 17 | ZEB1 |  |  | 717 | -0.317 | -0.3686 | Yes |
| 18 | PRDX1 |  |  | 719 | -0.326 | -0.3345 | Yes |
| 19 | RPL10 |  |  | 722 | -0.335 | -0.3008 | Yes |
| 20 | DLL3 |  |  | 736 | -0.414 | -0.2733 | Yes |
| 21 | HES6 |  |  | 752 | -0.523 | -0.2367 | Yes |
| 22 | RPS24 |  |  | 757 | -0.595 | -0.1773 | Yes |
| 23 | FABP5 |  |  | 759 | -1.641 | -0.0000 | Yes |
Table: GSEA details [plain text format]

  

Fig 2: RICHARDS\_NOWAKOWSKI\_SCIENCE\_2017\_IPC\_NEN3\_UPREG: Random ES distribution      
 Gene set null distribution of ES for **RICHARDS\_NOWAKOWSKI\_SCIENCE\_2017\_IPC\_NEN3\_UPREG**
